# The pro-atherogenic enzyme PAPP-A is active in eluates from human carotid and femoral atherosclerotic plaques

**DOI:** 10.1016/j.athplu.2024.09.001

**Published:** 2024-09-05

**Authors:** Mette Faurholdt Gude, Rikke Hjortebjerg, Mette Bjerre, Anne Kathrine Nissen Pedersen, Claus Oxvig, Lars Melholt Rasmussen, Jan Frystyk, Lasse Steffensen

**Affiliations:** a Medical/Steno Aarhus Research Laboratory, Department of Clinical Medicine, Aarhus University, Aarhus, Denmark; b Endocrine Research Unit, Department of Endocrinology, Odense University Hospital, Odense, Denmark; c Department of Clinical Research, Faculty of Health Sciences, University of Southern Denmark, Odense, Denmark; d Steno Diabetes Center Odense, Odense University Hospital, Odense, Denmark; e Dept. of Molecular Biology and Genetics, Aarhus University, Aarhus, Denmark; f Centre for Individualized Medicine in Arterial Diseases (CIMA), Odense University Hospital, Odense, Denmark; g Dept. of Clinical Biochemistry, Odense University Hospital, Odense, Denmark; h Dept. of Molecular Medicine, University of Southern Denmark, Odense, Denmark

**Keywords:** IGF1, IGFBP4, PAPP-A, STC2, Atherosclerosis

## Abstract

**Background:**

Pregnancy-associated plasma protein-A (PAPP-A) regulates bioavailability of insulin-like growth factor 1 (IGF1) in various tissues by proteolytic cleavage of a subset of IGF-binding proteins (IGFBPs). Pre-clinical studies have established a role of PAPP-A in atherosclerosis and proposed that targeting the proteolytic activity of PAPP-A has therapeutic value.

This study aimed to investigate whether human atherosclerotic plaques contain proteolytically active PAPP-A, a prerequisite for further considering PAPP-A as a therapeutic target in patients.

**Methods:**

We obtained carotid (*n* = 9) and femoral (*n* = 11) atherosclerotic plaques from patients undergoing vascular surgery and incubated freshly harvested plaque tissue in culture media for 24 h. Subsequently, conditioned media were assayed for PAPP-A, STC2, IGFBP4, and IGF1 using immunoassays. Enzymatic activity of PAPP-A was assessed by its ability to process recombinant IGFBP4-IGF1 complexes - a specific substrate of PAPP-A - by Western blotting.

**Results:**

PAPP-A and STC2 were detectable in conditioned media from both carotid and femoral plaques, with higher STC2 concentrations in eluates from carotid plaque incubations (*p* = 0.02). IGFBP4 and IGF1 were undetectable. Conditioned media from all 20 plaques exhibited PAPP-A proteolytic activity. However, no correlation between PAPP-A concentration and its proteolytic activity was observed, whereas the PAPP-A: STC2 molar ratio correlated with PAPP-A activity (R^2^ = 0.25, *p* = 0.03).

**Conclusion:**

This study provides evidence for the presence of enzymatically active PAPP-A in atherosclerotic plaques and underscores the need for further investigating potential beneficial effects associated with targeting PAPP-A in atherosclerotic cardiovascular disease.

## 1 Introduction

Pregnancy-associated plasma protein-A (PAPP-A) was originally identified as an antigen abundantly present in the circulation at late pregnancy [1], being secreted by the placental syncytiotrophoblasts [2], and today PAPP-A serves as a routine biomarker for fetal chromosomal abnormalities (e.g., Trisomy 21) [[3], [4], [5]].

In the 1990s, PAPP-A was shown to be expressed by various non-placental cells and to play a critical role in the regulation of the insulin-like growth factor (IGF) system [6]. PAPP-A belongs to the metzincin superfamily of metalloproteinases, but unlike other metalloproteinases, PAPP-A is unable to process extracellular matrix, and its only known substrates are a subset of IGF-binding proteins (IGFBPs), in particular, IGFBP4 [7]. PAPP-A binds to cell surfaces through glycosaminoglycan (GAG) [8], and thus, cleavage of IGFBP-4 occurs primarily within tissues. By liberating otherwise inactive IGF1 from IGBFP4 near the IGF1 receptor (IGF1R), PAPP-A serves as a key regulator of IGF activity [9,10]. The proteolytic activity toward IGFBP4 (but not other IGFBPs) is dependent on the three Lin12-Notch repeat (LNR) domains of PAPP-A, which enables specific targeting of PAPP-A [11,12]. Upon cleavage by PAPP-A, N-terminal (NT) and C-terminal (CT) fragments of IGFBP4 are generated, and their levels have been shown to correlate with PAPP-A concentration and reflect PAPP-A enzymatic activity [13].

Two paralogous proteins, stanniocalcin-1 and -2 (STC1 and STC2) have recently emerged as potent endogenous inhibitors of PAPP-A [14,15] operating in different tissue contexts in concert with PAPP-A to fine-tune IGF activity [14,16,17]. Proteolytically active PAPP-A is a 400 kDa (kDa) disulfide-bound homodimer and is inhibited by STC2 homodimers by the formation of a 500 kDa covalent heterotetrameric complex, the structure of which was recently delineated by cryo-electron microscopy [18]. STC1 does not inhibit PAPP-A covalently but is a competitive inhibitor with picomolar affinity [15]. A physiological relevance of the proposed STC2 - PAPP-A - IGFBP4 - IGF1 axis [19] was supported in a recent study showing that a genetic variant of STC2 with reduced PAPP-A inhibitory activity was linked to an up to 2 cm increase in human height [20].

Atherosclerosis is the focal build-up of lipid, fibrous tissue, and cells in the artery walls, and the underlying cause of heart attack and stroke [21]. Bayes-Genis et al. were the first to report the presence of PAPP-A in advanced coronary atherosclerotic plaques [22], and subsequent studies have confirmed this observation [16,23,24]. Circulating levels of PAPP-A and its enzymatic products, the IGFBP4 fragments, have also been shown to reflect atherosclerotic plaque burden and be candidate biomarkers of cardiovascular disease and mortality [[25], [26], [27]]. Pre-clinical studies have established an important role of murine PAPP-A in experimentally-induced atherosclerosis [24,28], first demonstrated by a remarkable 60–80 % reduction in lesion development in *Papp-a* knockout mice [29]. Subsequent pre-clinical studies have established *proof-of-principle* for inhibiting the proteolytic activity of PAPP-A in atherosclerosis either using recombinant STC2 [16] or an antibody specifically targeting the LNR domain of PAPP-A^30^. Whether this strategy has therapeutic value in human atherosclerosis remains unknown. Given that PAPP-A is primarily active within tissues, targeting PAPP-A to prevent atherosclerosis would require PAPP-A to be proteolytically active within the plaques. Accordingly, the objective of this study was to test the hypothesis that PAPP-A is present and proteolytically active in advanced human atherosclerotic lesions, as this is a crucial prerequisite for ongoing assessments regarding the potential therapeutic benefits of targeting PAPP-A in patients with atherosclerotic cardiovascular conditions.

## 2 Materials and methods

### 2.1 Human atherosclerotic plaque collection

We obtained atherosclerotic plaque tissue from patients undergoing endarterectomy on either the carotid or femoral artery due to ischemic stroke, transient ischemic attack (carotid artery) or peripheral arterial disease (femoral artery). All participants were treated with heparin prior to surgery. Immediately after excision, the plaque was placed in culture media, which consisted of Medium 199 (Sigma-Aldrich, Cat# M3769), supplemented with 25 mM HEPES (Sigma-Aldrich, Cat# H7523), 13 mM sodium hydrogen carbonate (Supelco, Cat# 1.06329), pH 7.4, 0.5 % human serum albumin (CSL Behring), 15 μM antipain (Sigma-Aldrich, Cat# A6191), 21 μM leupeptin (Sigma-Aldrich, Cat# L2884), penicillin (100 IU/mL) and streptomycin (100 IU/mL) (Cat#P4333 Sigma-Aldrich).

All participants gave their informed consent. The study was approved by the Central Denmark Region Committees on Health Research Ethics (1-10-72-278-18) and performed in compliance with the Helsinki Declaration.

Patient characteristics are shown in Table 1.

**Table 1:** 

	Carotid plaques (*n* = 9)	Femoral plaques (*n* = 11)
Sex (male)	3 (33 %)	7 (64 %)
Age (years)	70.4 ± 6.4	72.1 ± 5.0
Body mass index (kg/m^2^)	27.0 (1.9)	25.6 (3.6)
Diabetes	1 (11 %)	4 (36 %)
Smoking status (current/former/never)	2 (22 %)/4 (44 %)/3(33 %)	3 (27 %)/5 (45 %)/3 (27 %)
Antihypertensive medication	5 (56 %)	8 (73 %)
Antidiabetic medication	1 (11 %)	3 (27 %)
Lipid-lowering medication	4 (44 %)	10 (91 %)
Antiplatelet medication	5 (56 %)	8 (73 %)
Anticoagulant medication	1 (11 %)	2 (18 %)

Continuous data are displayed as mean ± SD.

Categorical data are presented as sums and percentages.

### 2.2 Plaque incubation

From each patient 275 mg plaque tissue was systematically segmented and placed in 24-well plates 1 mL of culture media was added pr well. The tissue was initially pre-incubated in culture media for 2 h, 37 °C to remove blood from the plaques, whereafter the plaque tissue was incubated in fresh culture media for 24 h at 37 °C. Hereafter, conditioned media corresponding to each plaque were pooled, frozen, and kept at −80 °C for subsequent analysis.

### 2.3 Histology and immunohistochemistry

Human carotid endarterectomy samples were harvested as described above, sliced in 4 mm thickness by razorblades, and immersion-fixed in 4 % formaldehyde in PBS for 24 h and subsequently embedded in paraffin. Five μm sections were stained by Mason trichrome at the Department of Pathology, Odense University Hospital. Immunohistochemistry for PAPP-A, IGFBP4, IGF1R, and STC2 was also performed at the Department of Pathology, Odense University Hospital. Demasking of antigens was done by 32 min treatment at 100 °C. The following dilutions of antibodies were used: IGFBP4 (Abcam, Cat# ab83846, diluted 1:75), PAPP-A (described previously [31], used at 5 μg/mL), STC2 (Abcam, Cat# ab255610, diluted 1:100), and IGF1R (R&D Systems, Cat# AF-305-NA, diluted 1:200). Detection was performed using the full-automated OptiView DAB IHC Detection Kit (8-8) (Ventana, Roche). Staining protocols conformed with standard practice of the Department of Pathology, Odense University Hospital, including negative controls (staining with indifferent antibodies and omission of primary antibodies), as well as staining of positive control multi-blocks with panels of human tissues, confirming relevant staining patterns at the used dilutions.

### 2.4 Immunoassays for PAPP-A and STC2 in conditioned media

PAPP-A and STC2 measurements were performed on conditioned media in duplicates by immunoassay kits from Anshlabs, Texas, USA (Cat# AL-101 for PAPP-A and Cat# AL-143 for STC2). Samples above the upper limit of detection were appropriately diluted and re-analysed. Similarly, attempts to measure IGF1 or IGFBP4 were done with assays from Anshlab (intact and total IGFBP4 (Cat# AL-128 and Cat# AL-126)) and IDS, iSYS (IGF1, Cat# IS-3900), however, no signals could be obtained.

### 2.5 PAPP-A activity in conditioned media

To examine if PAPP-A released from plaque tissue was biologically active and thereby could cleave exogenously added IGFBP4, we conducted a reaction test. Recombinant human IGFBP4 from RnD Systems (Cat# 804-GB-025) and IGF1 from Austral Biologicals (Cat# GF-050-8) were pre-incubated in media/PBS? and added to all conditioned media from different plaque?-incubations (200 μL) in a final concentration of 1200 ng/mL (of both IGF1 and IGFBP4). After 10 min incubation at room temperature, 100 μL was removed (the 0-h time-point) and added 3.3 mM EDTA, whereby the proteolytic activity of PAPP-A is terminated. The remaining 100 μL reacted for 24 h at 37 °C and was terminated by adding EDTA as above. Samples were immediately frozen and kept at −80 °C until analyses of intact IGFBP4 and CT-IGFBP4 by western blotting.

After the IGFBP4 reaction test, samples were boiled 95C in Laemmli buffer and loaded onto gels (CriterionTM TGX Stain-FreeTM, Precast Gels, Cat #5678085). The separated proteins were transferred to PVDF membranes (Trans-Blot Turbo, Midi Format, 0,2um, Cat#1704157) using Turbo Blot, Transfer System (BioRad). Membranes were blocked for 1 h in Tris-buffered saline with Tween20 (TBS-T) with 1 % BSA, then washed 3 times 5 min in TBS-T and incubated overnight with the primary antibody, Mab IBP185 (Hytest, Cat# 4IGF4) at 0.5 μg/mL, which binds CT-IGFBP4. Membranes were washed before adding a secondary antibody (Invitrogen, Cat# 31430, diluted 1:10,000). Blots were developed using Biorad ChemiDoc (ChemiDocTM MP Imaging System, BioRad), and analysed in Image Lab 6.1, BioRad. PAPP-A activity was defined as the intensity of the CT-IGFBP4 fragment band at 24 h relative to the starting point (intensity of the intact IGFBP4 band at 0 h) and normalized by dividing it with the tissue weight of the plaque sample. Media incubated for 24 h without plaque and subsequently undergoing the reaction test served as control.

### 2.6 In silico analysis

Single-cell RNA sequencing data from three independent studies of human plaque were analysed using Seurat v4 package [32] in R and annotated according to original studies [[33], [34], [35]]. UMAP and feature plots were generated by Seurat v4 package in R.

### 2.7 Statistics

Normality of data distribution was assessed using the *Shapiro-Wilk* test. For data that did not follow a normal distribution, we conducted a two-group comparison using the *Mann-Whitney* test, and comparison of four groups by Krustal-Wallis test followed by Dunn's multiple comparisons test. Linear regression analysis was conducted to assess the relationships between PAPP-A activity and either PAPP-A concentration or the PAPP-A:STC2 ratio. Statistical significance was defined as *p* < 0.05.

## 3 Results

### 3.1 Components of the STC2 - PAPP-A - IGFBP4 - IGF1 axis display similar expression patterns in human plaques

**Fig. 1:**
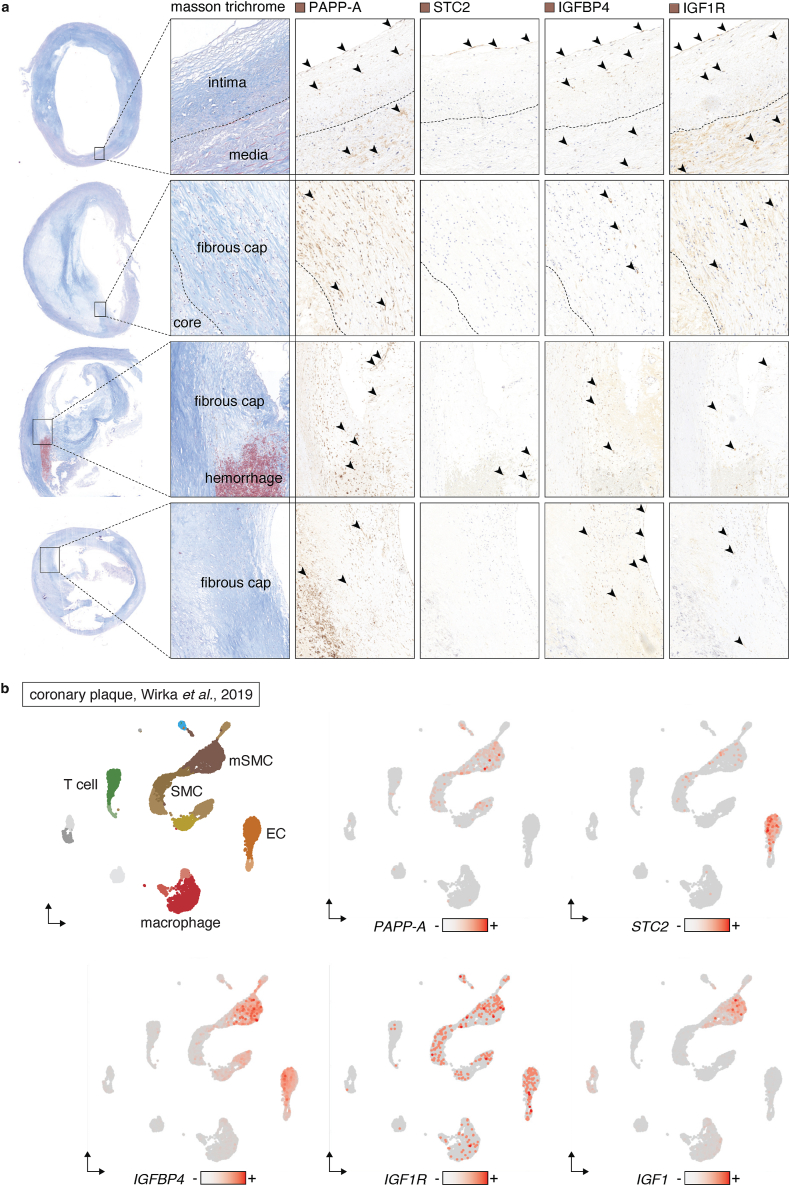
Expression pattern of components of the STC2 - PAPP-A - IGFBP4 - IGF1 axis. **a.** Carotid plaques stained by masson trichrome and immunohistochemistry for STC2, PAPP-A, IGFBP4, and IGF1R. Arrowheads point to examples of stained cells. **b.** Single-cell RNA sequencing data from Wirka et al., 2019, displayed as UMAPs showing annotated cell populations, and expression pattern of *PAPP-A*, *STC2*, *IGFBP4*, *IGF1R*, and *IGF1*. SMC = smooth muscle cell; mSMC = modulated SMC; EC = endothelial cell.

For PAPP-A to regulate IGF bioavailability in advanced plaques, PAPP-A would be expected to be present in the same regions as IGFBP4, IGF1, and IGF1R. To evaluate this, we stained for these components in serial sections from carotid and femoral plaques using immunohistochemistry (Fig. 1a).

In areas without plaque, PAPP-A was expressed in smooth muscle cells (SMCs) of the medial layer, intimal cells, and by endothelial cells (ECs). In plaque regions, PAPP-A expression was pronounced in cells of the fibrous cap (presumably SMC-derived cells), and in ECs of intraplaque vessels. Expression of IGFBP4 and IGF1R was less intense, but predominated in fibrous cap cells. In contrast, STC2 expression was only detected in ECs.

To evaluate the expression of PAPP-A and its associated genes at the transcriptional level, we leveraged three independent single-cell transcriptomic datasets from coronary and carotid plaques previously published [[33], [34], [35]] (Fig. 1b and Supplementary Figs. S1a–b). This analysis confirmed the immunohistochemical staining as *PAPP-A*, *IGFBP4*, and *IGF1R* were expressed by SMCs and modulated SMCs (presumably SMC-derived cells located in the intimal layer and the fibrous cap). Less consistently, *IGF1* was expressed by either modulated SMCs or macrophages depending on the dataset analysed. *IGFBP4* and *IGF1R* were also expressed by ECs, but this was not confirmed for *PAPP-A*. Validating the immunohistochemical staining, *STC2* expression was largely confined to ECs as well as cells associated with an intraplaque hemorrhage.

### 3.2 PAPP-A and STC2 were detectable in eluates of plaque tissue

**Fig. 2:**
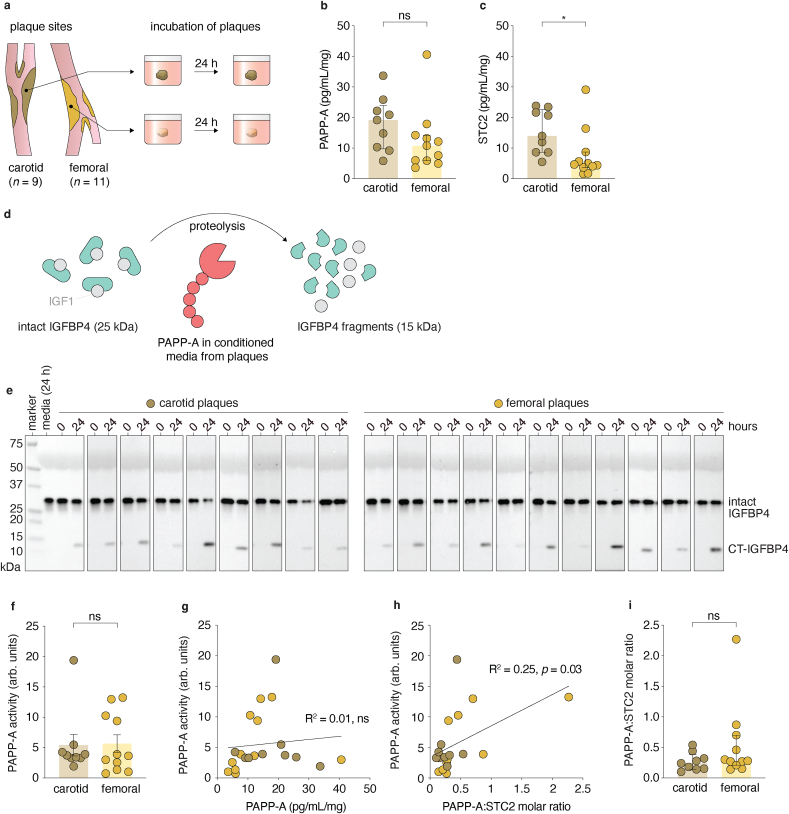
Proteolytically active PAPP-A is present in atherosclerotic plaques. **a.** Study design: Plaque samples from carotid and femoral arteries were incubated in culture media for 24 h, and conditioned media was harvested for analyses. **b-c.** PAPP-A (b) and STC2 (c) concentration in conditioned media after 24 h of incubation quantified by ELISA. **d.** Principle of PAPP-A activity assay: Recombinant IGFBP4:IGF1 complex (32 kDa) is incubated with sample containing active PAPP-A resulting in proteolytic cleavage to 14 kDa IGFBP4 fragments. **e.** Conditioned media from each of the 20 plaque samples were incubated with recombinant IGFBP4:IGF1 for 0 or 24 h and analysed for IGFBP4 cleavage by Western blotting. In all samples, no IGFBP4 fragments are detected at the 0-h time point, but IGFBP4 fragments emerges after 24 h. No PAPP-A activity was detected in the media without tissue incubation (media 24 h). **f.** Quantitation of PAPP-A activity based on Western blotting. **g.** Correlation between PAPP-A concentration and -activity. **h.** Correlation between PAPP-A:STC2 molar ratio and PAPP-A activity. **i.** PAPP-A:STC2 molar ratio in conditioned media.

To enable subsequent evaluation of PAPP-A activity in extracts from atherosclerotic plaques, we incubated plaques from carotid (*n* = 9) and femoral (*n* = 11) endarterectomies in culture media at 37 °C for 24 h. The conditioned media was used to determine the concentration of PAPP-A, STC2, IGFBP4, and IGF1 by immunoassays (Fig. 2a). IGFBP4 and IGF1 were not detected in any of the samples, but PAPP-A and STC2 were present in the conditioned media at quantifiable levels. Importantly, neither PAPP-A or STC2 were detectable in unconditioned culture media. PAPP-A tended to be higher in conditioned media from carotid plaques than femoral plaques: 19.2 (IQR: 10.3–22.2) vs. 10.8 (IQR: 8.7–21.7) pg/mL per mg tissue, *p* = 0.11) (Fig. 2b), while STC2 was significantly higher in carotid plaques than femoral plaques: 14.0 (IQR: 8.7–21.7) vs. 4.7 (IQR: 3.9–7.2) pg/mL per mg tissue, *p* = 0.016 (Fig. 2c). We observed no effect of sex for either type of plaque (Supplementary Figs. S2a–b).

### 3.3 PAPP-A is proteolytically active in eluates of atherosclerotic plaques

To assess PAPP-A activity in conditioned media from carotid and femoral plaques, conditioned media was incubated with recombinant IGFBP4-IGF1 complexes, a specific substrate of PAPP-A. The enzymatic activity of PAPP-A (Fig. 2d) was estimated as the intensity of CT-IGFBP4-fragment at 24 h relative to the amount of intact IGFBP4 at 0 h and normalized to plaque tissue weight [36]. IGFBP4 cleavage was detectable in every condition media investigated from both carotid and femoral plaques, demonstrating that the plaques were able to secrete proteolytically active PAPP-A when incubated *in vitro* (Fig. 2e). Importantly, unconditioned culture media did not display any IGBFP4 cleavage after 24 h of incubation.

There was no difference in PAPP-A activity in conditioned media from carotid- and femoral plaques (Fig. 2f), and PAPP-A activity did not correlate with the concentration of PAPP-A measured by immunoassay in the same conditioned media sample (R^2^ = 0.01, *p* = 0.67) (Fig. 2g). In contrast, we found a positive correlation between PAPP-A activity and the immunoassay-detected PAPP-A:STC2 molar ratio (R^2^ = 0.25, *p* = 0.03) (Fig. 2i), whereas the molar ratio of PAPP-A:STC2 did not differ between anatomical sites (Fig. 2h). No effect of sex for either type of plaque was observed (Supplementary Figs. S2c–d).

## 4 Discussion

The objective of this study was to test the hypothesis that PAPP-A exhibits proteolytic activity in advanced human atherosclerotic plaques, a prerequisite for the potential therapeutic targeting of PAPP-A in human atherosclerosis. Indeed, we confirmed the hypothesis by showing that plaques when incubated ex vivo release enzymatically active PAPP-A, irrespective of whether the plaque originates from the carotid or the femoral artery. We also demonstrated by immunohistochemistry and single-cell RNA sequencing datasets that PAPP-A is present in human atherosclerotic lesions, as demonstrated in previous studies, and furthermore revealed the expression of all components of the STC2-PAPP-A-IGFBP4-IGF1 axis within the same plaque regions and cell populations. This indicates that PAPP-A actively participates in regulating IGF1 bioavailability in advanced human plaques *in vivo*.

Our findings agree with numerous previous observations. PAPP-A appears to exert its function primarily at local sites, and levels within tissues and extravascular fluids can be several-fold higher than those of the circulation [13,37,38]. This suggests that PAPP-A exerts its biological role in the local microenvironment. Noteworthy, the local PAPP-A effect may have clinical importance, as elevated circulating concentrations of PAPP-A and IGFBP-4 fragments have been associated with an increased cardiovascular risk and mortality [26,27].

The idea of considering PAPP-A as a therapeutic target in atherosclerosis first emerged from its abundant expression in coronary plaques that had ruptured or eroded in patients, who experienced sudden cardiac death [22]. The concept gained traction through a series of pre-clinical studies illustrating that genetic deletion [29] or impairment [39] of murine PAPP-A significantly reduced lesion development in mouse models. Subsequent studies provided direct proof-of-principle of therapeutic targeting of PAPP-A's proteolytic activity, as plaque lesion development was reduced by treatment with recombinant STC2 [16] or inhibitory PAPP-A antibodies [30]. A commonality among all pre-clinical studies is the initiation of intervention during lesion development, a phase characterized by extensive cell proliferation, likely in part driven by IGF signaling. However, the extent to which the IGF system operates in advanced human lesions has not been investigated. Our findings support that plaques contain proteolytically active PAPP-A, that can be released by ex vivo incubation, and that PAPP-A associates with other components of the IGF-system within plaque tissue suggesting that the IGF-system has a role even in late stages of atherosclerosis within a therapeutically relevant window. However, whether inhibition of the IGF system at this stage of plaque development is beneficial in terms of plaque stabilization or regression necessitates direct investigation, e.g., by anti-PAPP-A treatment after initiation of lesions.

Our study has limitations that should be acknowledged. The described experiments were based on incubating freshly harvested plaque tissue in culture media to enable functional investigation of native PAPP-A from the plaque tissue. As PAPP-A binds to GAGs in the tissue via its SCR3 and SCR4 domains [8], PAPP-A does not readily diffuse into the culture medium, and our investigations are likely limited to only a fraction of the PAPP-A present in the tissue. Another issue is heparin, which was administered intravenously to patients prior to surgery. Heparin administration mice [40], pigs [41], and humans [42] causes marked elevations in circulating PAPP-A (presumable by outcompeting its binding to tissue heparan sulfate (HS) as this effect of heparin has been observed for numerous HS-binding proteins [43]). Therefore, the administration of heparin to patients before surgery might have influenced the amount of PAPP-A released into the culture medium, but if so, this may have reduced rather than increased the release of PAPP-A from the plaques during incubation.

IGF1 and IGFBP4 were undetectable in conditioned media. Rapid IGF1 turnover in our *in vitro* conditions may explain our inability to detect IGF1. The absence of immunoreactive IGFBP4 in the culture media remains unexplained; it could relate to tight binding to the extracellular matrix.

In the study, we observed a moderate correlation between PAPP-A activity and the PAPP-A:STC2 molar ratio based on immunoassays, but no correlation between PAPP-A activity and PAPP-A concentration. This suggests that a portion of the PAPP-A in the culture medium is inhibited by STC2. However, our experimental setup does not allow us to distinguish between a scenario where the STC2-PAPP-A complex formed in the plaque tissue (as previously proposed [16]), or in the culture medium during the 24 h of incubation. We observed a 4:1 M ratio between STC2 and PAPP-A in the conditioned media after 24 h. Given that STC2 forms an irreversible covalent complex with PAPP-A, prolonged incubation would likely completely shut down PAPP-A activity. Consequently, our estimation of active PAPP-A released from plaque tissue is likely underestimated since a fraction of it would be inhibited by STC2 during the 24-h incubation period. However, these limitations would bias our results toward underestimating both the amount of PAPP-A in human plaques and PAPP-A activity.

In conclusion, we show that PAPP-A is proteolytically active toward its substrate IGFBP4:IGF1 in conditioned media from all 20 human plaques investigated from two different anatomical sites. This finding further substantiates the role of the STC2 - PAPP-A - IGFBP4 - IGF1 axis in atherosclerosis and encourages additional studies of PAPP-A as a therapeutic target.

## Declaration of competing interest

The authors declare that they have no known competing financial interests or personal relationships that could have appeared to influence the work reported in this paper.

## Appendix A Supplementary data

Multimedi component 1 Supplementary. Expression pattern of components of the STC2 - PAPP-A - IGFBP4 - IGF1 axis. **a-b.** Single-cell RNA sequencing data from Pan et al., 2020 (a), and Alsaigh et al., 2020 (b), displayed as UMAPs showing annotated cell populations, and expression pattern of *PAPP-A*, *STC2*, *IGFBP4*, *IGF1R*, and *IGF1*. SMC = smooth muscle cell; mSMC = modulated SMC; EC = endothelial cell. Supplementary Figure S2. Sex stratification of plaque eluate measurements. **a-b.** PAPP-A (a) and STC2 (b) concentration in conditioned media after 24 h of incubation quantified by ELISA stratified by sex. **c.** Quantitation of PAPP-A activity based on Western blotting stratified by sex. **d.** PAPP-A:STC2 molar ratio in conditioned media stratified by sex. f = female, m = male. Multimedi component 1
