# Supplementary figures and images for: The pro-atherogenic enzyme PAPP-A is active in eluates from human carotid and femoral atherosclerotic plaques
Source: Atheroscler Plus. 2024 Sep 5;57:30–6. doi: 10.1016/j.athplu.2024.09.001 (PMC11415872; doi:10.1016/j.athplu.2024.09.001)

**a** carotid plaque, Pan *et al.*, 2020

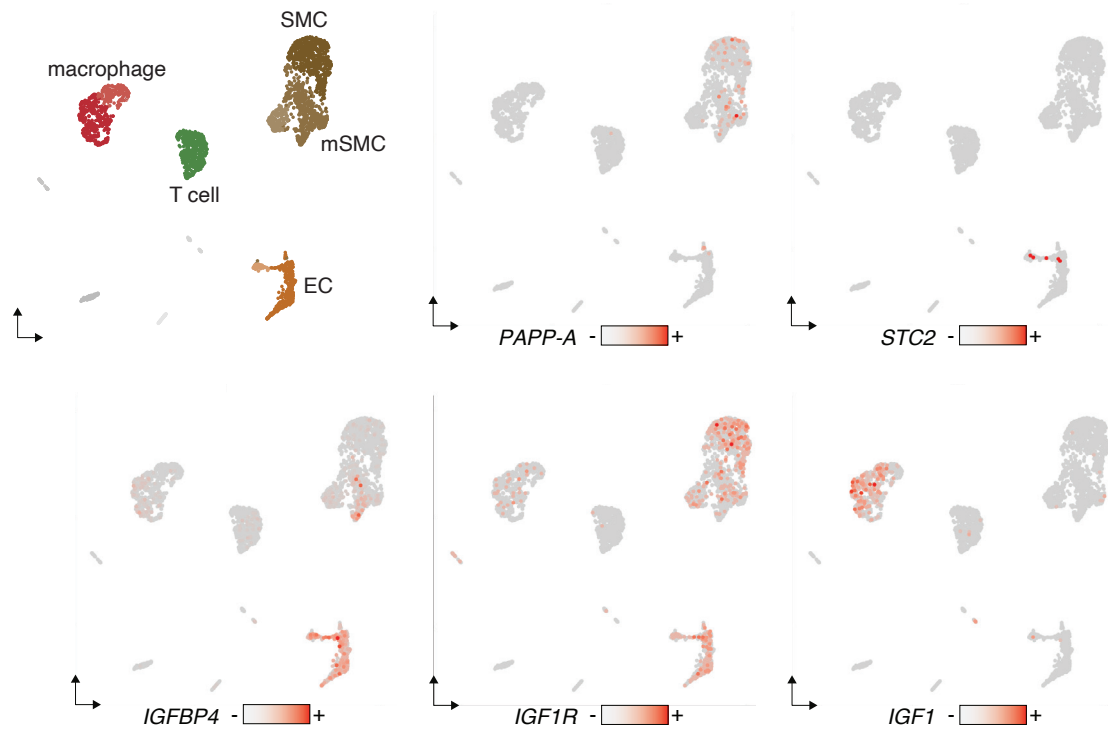

**b** carotid plaque, Alsaigh *et al.*, 2020

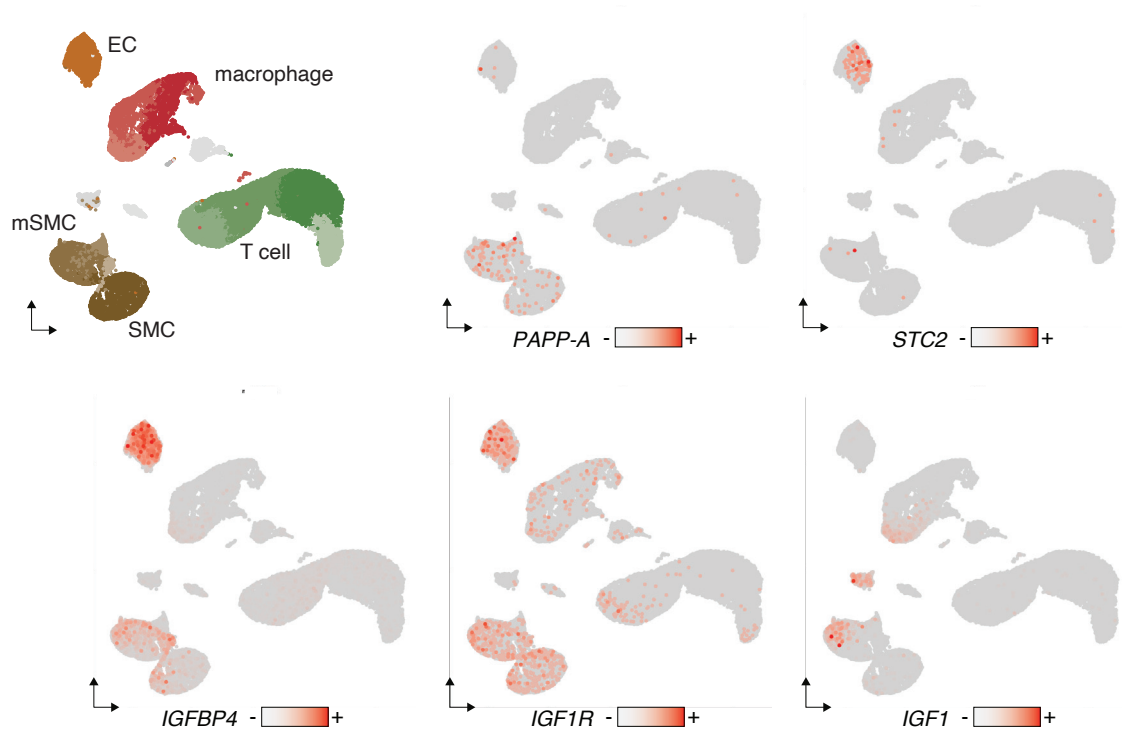

supplementary figure S2

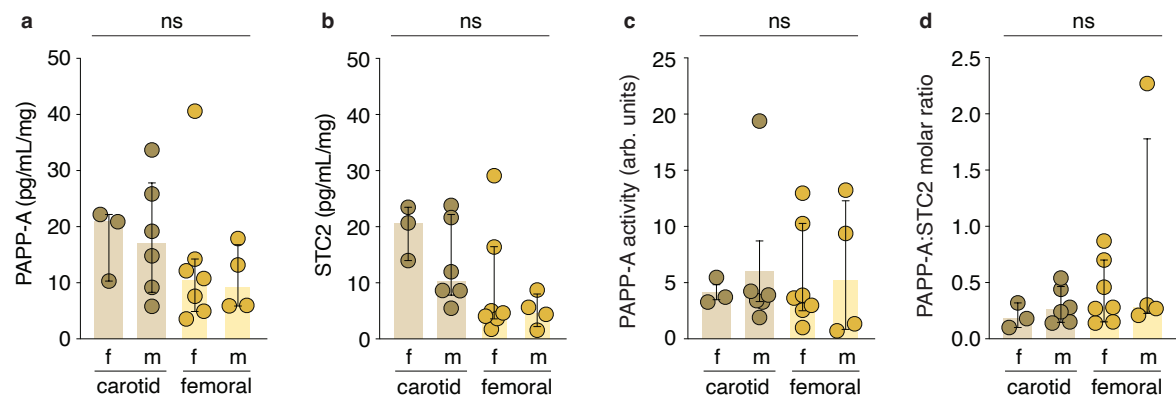

Supplement: Multimedi component 1 — Supplementary. Expression pattern of components of the STC2 - PAPP-A - IGFBP4 - IGF1 axis. a-b. Single-cell RNA sequencing data from Pan et al., 2020 (a), and Alsaigh et al., 2020 (b), displayed as UMAPs showing annotated cell populations, and expression pattern of PAPP-A, STC2, IGFBP4, IGF1R, and IGF1. SMC = smooth muscle cell; mSMC = modulated SMC; EC = endothelial cell. Supplementary Figure S2. Sex stratification of plaque eluate measurements. a-b. PAPP-A (a) and STC2 (b) concentration in conditioned media after 24 h of incubation quantified by ELISA stratified by sex. c. Quantitation of PAPP-A activity based on Western blotting stratified by sex. d. PAPP-A:STC2 molar ratio in conditioned media stratified by sex. f = female, m = male. [file mmc1.pdf]
